# β-Boswellic Acid Suppresses Breast Precancerous Lesions *via* GLUT1 Targeting-Mediated Glycolysis Inhibition and AMPK Pathway Activation

**DOI:** 10.3389/fonc.2022.896904

**Published:** 2022-05-31

**Authors:** Fengjie Bie, Guijuan Zhang, Xianxin Yan, Xinyi Ma, Sha Zhan, Yebei Qiu, Jingyu Cao, Yi Ma, Min Ma

**Affiliations:** ^1^School of Traditional Chinese Medicine, Jinan University, Guangzhou, China; ^2^School of Nursing, Jinan University, Guangzhou, China; ^3^The First Clinical Medical College, Southern Medical University, Guangzhou, China; ^4^The Oncology Department, The First Affiliated Hospital of Jinan University, Guangzhou, China; ^5^Department of Cellular Biology, Institute of Biomedicine, National Engineering, Research Center of Genetic Medicine, Key Laboratory of Bioengineering Medicine of Guangdong Province, The National Demonstration Center for Experimental Education of Life Science and Technology, Jinan University, Guangzhou, China

**Keywords:** β-boswellic acid, breast precancerous lesions, glycolysis, GLUT1, AMPK

## Abstract

Breast carcinoma is a multistep progressive disease. Precancerous prevention seems to be crucial. β-Boswellic acid (β-BA), the main component of the folk medicine *Boswellia serrata* (*B. serrata*), has been reported to be effective in various diseases including tumors. In this work, we demonstrated that β-BA could inhibit breast precancerous lesions in rat disease models. Consistently, β-BA could suppress proliferation and induce apoptosis on MCF-10AT without significantly influencing MCF-10A. Kyoto Encyclopedia of Genes and Genomes (KEGG) analysis suggested that β-BA may interfere with the metabolic pathway. Metabolism-related assays showed that β-BA suppressed glycolysis and reduced ATP production, which then activated the AMPK pathway and inhibited the mTOR pathway to limit MCF-10AT proliferation. Further molecular docking analysis suggested that GLUT1 might be the target of β-BA. Forced expression of GLUT1 could rescue the glycolysis suppression and survival limitation induced by β-BA on MCF-10AT. Taken together, β-BA could relieve precancerous lesions *in vivo* and *in vitro* through GLUT1 targeting-induced glycolysis suppression and AMPK/mTOR pathway alterations. Here, we offered a molecular basis for β-BA to be developed as a promising drug candidate for the prevention of breast precancerous lesions.

## 1 Introduction

The latest research shows that female breast cancer has surpassed lung cancer as the most commonly diagnosed (11.7%) and the fifth most lethal (6.9%) cancer in the world (1). Due to insufficient financial and medical resources, the sharply increasing incidence of female breast cancer results in enormous social and economic burden not only to individuals but also to nations. Breast carcinogenesis is a multistep, multipath, and multiyear disease of progressive gene-associated tissue damage (2). The positive interventions to arrest these steps may impede or delay canceration. Therefore, efforts to develop precancerous therapy and prevention seem to be extremely important.

Currently, no publicly recognized breast precancerous therapy has been achieved. Tamoxifen (Tam) as the most effective and widely used antiestrogen therapy is applied to reduce the incidence of breast cancer in high-risk women. Due to its principal toxicities like endometrial cancers and thromboembolic events (3), USPSTF announced that Tam was not suggested for prevention in non-high-risk populations. An effective and well-tolerated remedy is urgently needed to fill up the therapeutic gap between precancerous lesion and breast cancer. The Warburg effect is one of the main characteristics of cancer, which describes a phenomenon in which cancer cells metabolize more glucose by glycolysis than normal tissues despite the presence of oxygen, producing substantial lactate. There are lines of evidence that showed that cell metabolism may be reprogrammed at the precancerous stage with the involvement of glycolysis increase (4, 5), which created a potential therapeutic window between normal tissues and premalignant lesions. Glucose transporters (GLUTs) play critical roles in the alteration of metabolism. GLUTs are membrane proteins regulating glucose uptake and maintaining intracellular glucose concentration, which act on the upstream of glycolysis (6). Glucose transport 1 (GLUT1) is upregulated in many types of cancers. Targeting GLUT1 could reduce glucose uptake and induce apoptosis in cancer cells (7–9). Some small molecules derived from natural compounds have been proven to be GLUT1 inhibitors with anti-cancer effects (10–12).

Nowadays, about 50% of pharmaceuticals are derived from natural compounds and their derivatives. Phytochemicals such as flavonoids, alkaloids, and diterpenoids function well in diverse fields with higher safe dose and fewer side effects (13). Boswellic acids (BAs) are pentacyclic triterpenes, the main active components of *Boswellia serrata* Roxb. BAs and their derivatives have been proven to possess significant potential in treating inflammation diseases concerning asthma, rheumatoid arthritis, hydrocephalus, and, more importantly, a variety of cancers. The results of several studies indicated that BAs exert suppressing actions against tumor survival, proliferation, angiogenesis, and metastasis with outstanding tolerance in the past decades (14), while the potential mechanism of BAs on treating breast precancerous lesions is still elusive.

In this article, we found that β-BA could prevent precancerous lesions in rat models, and exert an inhibition effect on MCF-10AT. Pathway enrichment indicated that the effect of β-BA may focus on the metabolism. Further experiment showed that the glycolysis process of MCF-10AT was inhibited *via* targeting GLUT1. Our work may provide evidence for the potential clinical use of β-BA in breast precancerous prevention.

## 2 Materials and Methods

### 2.1 Cell Culture

The MCF-10A and MDA-MB-231 cell lines were purchased from the American Type Culture Collection (ATCC) and cultured according to the manufacturer’s directions. MCF-10AT was obtained from the Barbara Ann Karmanos Cancer Institute. MCF-10A was derived from spontaneous immortalized breast epithelial cells of a patient with fibrocystic disease. MCF-10AT was obtained by H-ras-transfected MCF-10A cells. MDA-MB-231 was an estrogen receptor-negative breast cancer cell line. MCF-10A and MCF-10AT were maintained in DMEM/F12 medium supplemented with 10% horse serum, 20 ng/ml EGF, 10 μg/ml insulin, and 50 μg/ml hydrocortisone. MDA-MB-231 was cultured with Leibovitz’s L-15 medium containing 10% FBS. All cells were incubated in the 37°C incubator with 5% CO_2_ and 95% humidity.

### 2.2 Colony Formation Assay

Cells were seeded into six-well plates at the concentration of 500 cells per well and maintained with or without β-BA for 2 weeks. For analysis, the plates were washed with PBS twice, fixed with methanol for 30 min, stained by 0.1% crystal violet, and then counted *via* ImageJ.

### 2.3 Cell Viability Assay

The effect of β-BA on cells was assessed by CCK8 assay. Twenty-four hours before treatment, 4 × 10^3^ cells were seeded into each well, followed by adding various concentrations of β-BA. On the day of detection, the supernatant was replaced by 10% CCK8 mixture (10 μl of CCK8 with 90 μl of basic medium for each well). With additional 2 h incubation at 37°C, the plate was read at 450 nm by a microplate spectrophotometer.

### 2.4 Apoptosis Assay by Flow Cytometry

The apoptosis assay was determined using an Annexin V-APC/PI detection kit (DOJINDO, Japanese) following the manufacturer’s protocol. The cells under detection were harvested, washed, and suspended in the binding buffer. After staining with the Annexin V/PI staining mixture for 15 min at room temperature, the samples were analyzed by flow cytometry (Beckman, USA) within 1 h.

### 2.5 Target Prediction and KEGG Enrichment Analysis of β-BA

To identify potential targets and signaling pathways, β-BA (PubChem CID:168928) was submitted to Bioinformatics Analysis Tool for Molecular mechanism of TCM (BATMAN-TCM, http://bionet.ncpsb.org/batman-tcm/). The predicted targets were submitted for Kyoto Encyclopedia of Genes and Genomes (KEGG) analysis on the DAVID website to enrich related signaling pathways.

### 2.6 ECAR Detection

Extracellular acidification rate (ECAR) was analyzed using Glycolysis Stress Test Kit *via* Seahorse XF96 instrument (Agilent Technologies, USA). Briefly, cells were pre-treated with or without β-BA for 48 h and then seeded into a plate one day before testing. During the experiment, cells were treated with glucose, oligomycin, and 2-deoxy-glucose sequentially with ECAR value quantified at different time points.

### 2.7 2-NBDG Glucose Uptake Detection

2-NBDG (Med Chem Express, USA), a fluorescent D-glucose analog, was used to detect glucose uptake level of cells following treatment with β-BA. The stock solution was diluted with basic medium to yield 20 μM working solution in advance. After treatment with 40 μM β-BA for 48 h, 2-NBDG working solution was added to cover the surface of culture cells sufficiently and incubated at 37°C for 30 min. Then, the cells were washed with PBS twice to remove residual reagent and detected as soon as possible. The intensity of fluorescence was detected using flow cytometry (Beckman, USA) with FL1.

### 2.8 Western Blot Assay

For cells, reasonable RIPA lysis buffer was added to the dishes following twice washing with PBS. Then, cells were harvested by gentle scraping and stored at −80°C if not processed immediately. The well-prepared total protein was separated by 12% or 10% SDS-PAGE and transferred to a 0.22-μm polyvinylidene fluoride (PVDF) membrane (Millipore, Billerica, USA). After blocking with 5% skimmed milk at room temperature, the membrane was washed 3 times and incubated with a certain first antibody overnight at 4°C. HRP-conjugated secondary antibody (Cell Signaling Technology, Inc., Boston, USA) was used with bands visualized by the ECL detection kit.

### 2.9 Real-Time PCR Assay

RNA was extracted from control and drug-treated cells using Trizol reagent (Invitrogen, Carlsbad, CA, USA) and reverse transcribed (Takara, Japan). Then, the cDNA was submitted to quantitative PCR using 2× SYBR green master mix (Bio-Rad laboratories, USA) on a CFX96 detection system according to the manufacturer’s instructions. Data of samples were normalized by the levels of ACTB. The sequences of primers were acquired from PrimerBank.

### 2.10 Plasmid Construction and Transfection

The CDS sequence of GLUT1 was successfully cloned into pcDNA3.1 plasmid and validated by sequencing. Then, the plasmid pcDNA3.1-GLUT1 and its control were transfected into MCF-10AT using Lipofectamine 2000 (Invitrogen, CA, USA) according to the protocol of the manufacturer. Twenty-four hours after transfection, the cells were treated with vehicle or β-BA 48 h before downstream assays.

### 2.11 Preparation of DMBA Solution

DMBA was precisely weighted, dissolved in sesame oil, and ultrasonic processed in a water bath at a constant temperature of 60°C to get a homogeneous mixture. The concentration of DMBA was 7 mg/ml.

### 2.12 Preparation of Tamoxifen Cream

The blank matrix was heated to melt and slowly mixed with Tam *via* constant stirring. Then, the mix was cooled down to room temperature. The concentration of Tam in cream was 5 mg/g.

### 2.13 Preparation of Blank Matrix and Beta-Boswellic Acid Cream

The blank matrix was prepared as previously described. Briefly, the water phase was prepared by dissolving 7 g of KOH in an appropriate amount of distilled water and heated to 80°C. Stearic acid (140 g) was heated, melted, and cooled down to 80°C. KOH water solution (100 g) and glycerol were added and stirred constantly. Then, an appropriate amount of distilled water was added, and the volume was metered to 1,000 ml to obtain the blank matrix (Mat). The matrix was continuously stirred and cooled down to 40°C. Then, a definite dose of β-BA (MedChemExpress, USA) was added, thoroughly mixed, and cooled down to room temperature to obtain the β-BA cream. The concentration of β-BA in cream was 5 mg/g.

### 2.14 Establishment and Treatment of the Rat Model With Precancerous Breast Lesions

Forty female SD rats that were 6 weeks old were fed in the SPF-grade fostering environment of Jinan University Animal Center. Then, the rats were randomly divided into four groups (10 per group): the vehicle group, the disease model group, the Tam-treated group, and the β-BA acid-treated group. One-week post arrival, the hair around the breast (1 cm diameter) of these rats was removed by depilatory paste for follow-up treatment. Finally, the rats were sacrificed and tested in the 14th week. This animal experiment was approved by the Laboratory Animal Ethics Committee of Jinan University. The experimental procedure and animal welfare strictly followed the Care and Use of Laboratory Animals (Ministry of Science and Technology of China, 2006) and related regulations of Jinan University.

#### 2.14.1 Establishment of the Breast Precancerous Rat Model

The breast precancerous lesion was induced by the combination of DMBA, estrogen, and progesterone as described previously. Briefly, the rats received DMBA sesame oil (1 ml/100 g body weight) *via* gavage and benzoate estradiol (0.5 mg/kg body weight) *via* intramuscular injection from Day 1 to Day 3. Then, progesterone (4 mg/kg body weight) was injected on Day 4. The rats were observed on Day 5. Continuous 12 cycles (5 days per cycle) were performed.

#### 2.14.2 Therapeutic Intervention of Tamoxifen

From Day 1 of model establishment to the end of the 14th week, 0.2 g of Tam cream (5 mg/g cream) was smeared with cotton swabs on the exposed skin of breast of each rat once a day.

#### 2.14.3 Therapeutic Intervention of Beta-Boswellic Acid

From Day 1 of model establishment to the end of the 14th week, 0.2 g of β-BA cream (5 mg/g cream) was smeared with cotton swabs on the exposed skin of breast of each rat once a day.

### 2.15 Hematoxylin and Eosin Staining

Once separated from the bodies of rats, organ tissues were fixed in formalin immediately, and later embedded in paraffin. The blocks were cut into 5-μm sections. Before staining, the slides were baked, dewaxed, and hydrated. For HE staining, the slides were stained with hematoxylin first for 2 min followed by hydrochloric acid alcohol differentiation and then eosin stain.

### 2.16 Statistical Analysis

Error bars represent the S.D., as indicated in figure legends. All statistical tests were analyzed by Student’s two-tailed *t*-test for comparison of two groups and by analysis of variance (with post-hoc comparisons using Dunnett’s test) for comparison of multiple groups using GraphPad Prism v. 8.0 (La Jolla, CA). *p* < 0.05 was considered statistically significant.

## 3 Results

### 3.1 β-BA Suppresses Cell Growth With Different Effects

**Figure 1:**
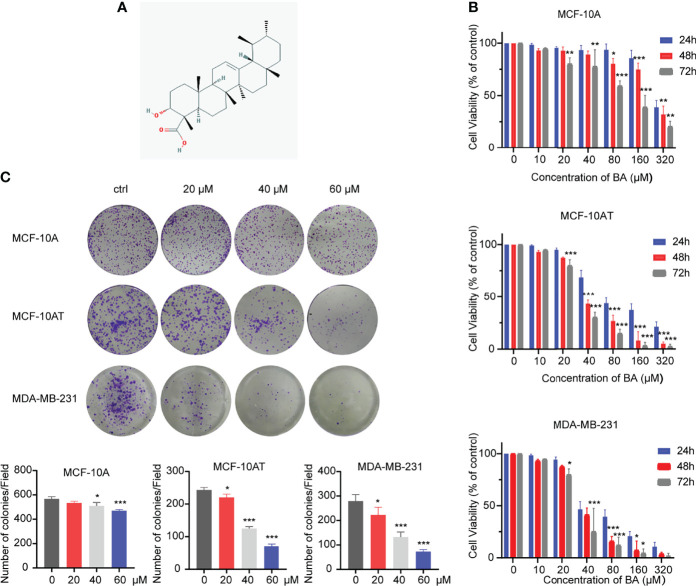
β-BA inhibits cell proliferation in MCF-10AT and MDA-MB-231. **(A)** Chemical structure of β-BA. **(B)** Cell viability was detected by CCK8 assay following treatment with different doses of β-BA. Cells were treated with 0, 10, 20, 40, 80, 160, and 320 μM of β-BA for 24, 36 and 48 h, respectively. IC_50_ values for each cell line at different time points were determined. **(C)** Colony formation assay for MCF-10A, MCF-10AT, and MDA-MB-231 treated with 0, 20, 40, and 60 μM of β-BA, respectively. Numbers of colonies were counted and shown below. *p < 0.05, **p < 0.01, and ***p < 0.001.

The chemical structure of cordycepin is shown in **Figure 1A**. Cell lines used in this experiment involve MCF-10A, MCF-10AT, and MDA-MB-231. Due to the various similarities between precancerous lesions and tumors, a commonly used triple-negative breast cancer cell line, MDA-MB-231, was used as a control. These cells were treated with β-BA at various concentrations for 24, 48, or 72 h to investigate the toxic effect of β-BA on these cells. As shown in **Figure 1B**, the CCK-8 assay showed that β-BA displayed a significant inhibition effect on MCF-10AT and MDA-MB-231, and a mild effect on MCF-10A. The IC_50_ values of β-BA at 48 and 72 h were 89.04 and 91.41 μM in MCF-10A cells, 37.2 and 30.3 μM in MCF-10AT cells, and 39.4 and 26.23 μM in MDA-MB-231 cells, respectively. In addition, a colony formation assay was performed to assess the inhibitory effect of β-BA. The results revealed that MCF-10AT and MDA-MB-231 treated with 5, 10, and 20 μM β-BA for 2 weeks formed fewer and smaller colonies than control (**Figure 1C**), indicating that β-BA inhibited the growth of these cells in a dose-dependent manner, while MCF-10A was suppressed in a relatively milder way.

### 3.2 β-BA Promotes Apoptosis in MCF-10AT

**Figure 2:**
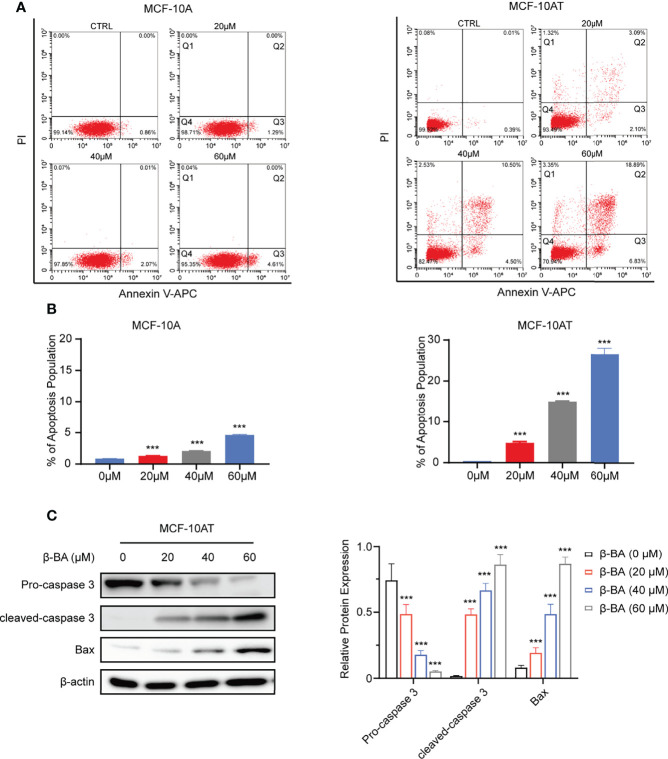
β-BA promotes apoptosis in MCF-10AT. **(A)** FACS analysis Annexin V/PI staining was used to determine apoptosis induced by β-BA. **(B)** The population of early apoptosis and that of late apoptosis were counted. **(C)** The expression levels of cleaved-caspase 3, pro-caspase 3, and Bax were analyzed in MCF-10AT following a 48-h treatment with β-BA at designated concentrations. Protein levels were quantified by Image J and normalized with that of β-actin in the right panel. Data were shown as the mean ± S.D from independent experiments. ***p < 0.001.

We further explored whether β-BA had an effect on the apoptosis of MCF-10A and MCF-10AT. After treating with β-BA for 48 h, MCF-10A and MCF-10AT cells were analyzed *via* Annexin V/PI staining. As shown in **Figures 2A, B**, the Q3 area indicating early apoptosis and the Q2 area indicating late apoptosis were both dramatically increased for MCF-10AT, suggesting that β-BA induced significant apoptosis, while β-BA induced apoptosis on MCF-10A in a relative milder way. To further confirm these results, we performed Western blot to examine protein expression of apoptosis-related genes. Activation of cysteine-aspartic acid proteases (caspases) plays an important role in cell apoptosis. As shown in **Figure 2C**, the cleaved form of caspase 3 was dominantly increased in MCF-10AT. Bax, the core regulator of the intrinsic pathway of apoptosis, also increased following β-BA treatment in MCF-10AT. Altogether, β-BA markedly promoted apoptosis in MCF-10AT with a relatively milder effect on MCF-10A.

### 3.3 β-BA Suppresses Glycolysis and Activates AMPK Pathway in MCF-10AT

**Figure 3:**
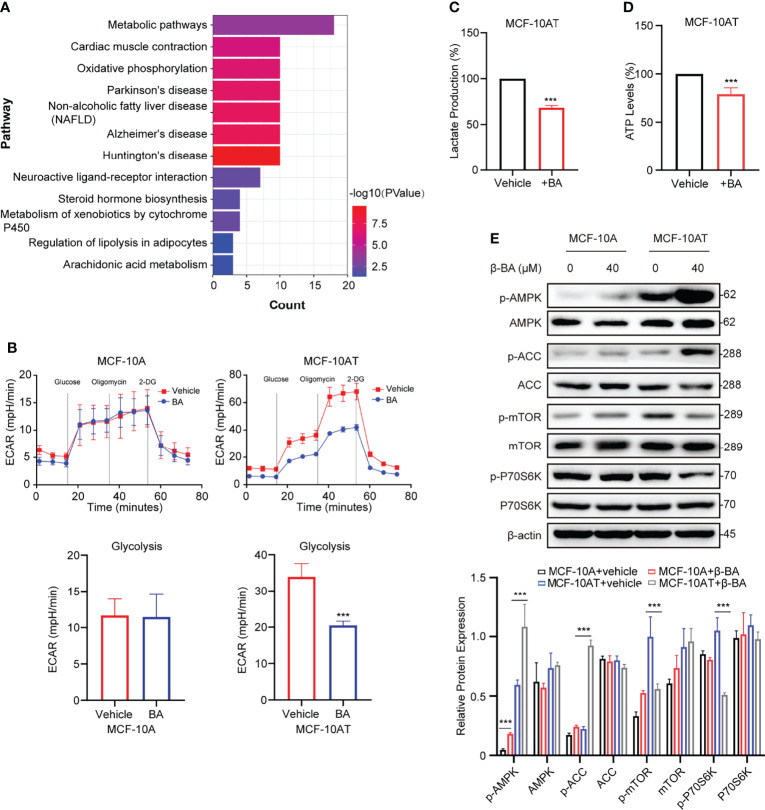
β-BA suppresses glycolysis and activates the AMPK pathway in MCF-10AT. **(A)** KEGG pathway enrichment analysis was performed with predicted targets derived from BATMAN-TCM. **(B)** The cellular ECAR was measured in MCF-10A and MCF-10AT cells with or without β-BA treatment. Statistics of glycolysis ECAR values are shown in the lower panel. The lactate production **(C)** and ATP levels **(D)** were measured in MCF-10AT cells with or without β-BA treatment. **(E)** The expression levels of AMPK, p-AMPK, ACC, p-ACC, mTOR, p-mTOR, p70S6k, and p-p70S6k were analyzed in MCF-10A and MCF-10AT following a 48-h treatment with 40 μM β-BA. Protein levels were quantified by ImageJ and normalized with that of β-actin in the lower panel. Data were shown as the mean ± S.D from independent experiments. ***p < 0.001.

To further explore the potential mechanism of β-BA in inhibiting proliferation and inducing apoptosis on MCF-10AT, we searched the BATMAN-TCM website to predict potential targets. A total of 45 genes were obtained, including COX1, ADH1B, and CYP17A1. To further characterize the potential affected pathways, these genes were submitted for KEGG analysis. The top enriched pathway was the metabolic pathway (**Figure 3A**). Considering glycolysis increase is a key character of precancerous lesions compared to normal tissues, and we detected glycolysis changes of MCF-10A and MCF-10AT *via* seahorse assays. Following treatment with β-BA, MCF-10AT displayed decreased extracellular acidification rate (ECAR) compared to control cells, while the change of ECAR in MCF-10A was mild (**Figure 3B**). Consistently, decreased lactate production was observed in β-BA-treated MCF-10AT (**Figure 3C**), and the ATP level was also decreased (**Figure 3D**). Since ATP is the main source of energy required by most *in vivo* biochemical processes, its reduction may limit cell survival. The AMPK system is a key player in regulating energy balance, which could be activated by increased AMP/ATP ratio. Once activated, it switches anabolic processes to catabolic pathways. Here, we found that the phosphorylation of AMPK α-subunit was enhanced following β-BA treatment with the total expression of AMPK not influenced. The activation of AMPK downstream target ACC further confirmed these. mTORC1 as a reported target of AMPK functions to promote cell growth and proliferation. Here, the results of Western blot showed that p-mTOR as well as the downstream target p-p70S6k of MCF-10AT were reduced following β-BA treatment, with the pan-protein expression not affected (**Figure 3E**). Altogether, β-BA could suppress the glycolysis process and induce lactate and ATP reduction in MCF-10AT. The energy stresses would activate the AMPK pathway and inhibit the mTORC1 pathway, which may partially explain the inhibition effects of β-BA on MCF-10AT.

### 3.4 β-BA Interacts With GLUT1 to Block Glucose Uptake in MCF-10AT

**Figure 4:**
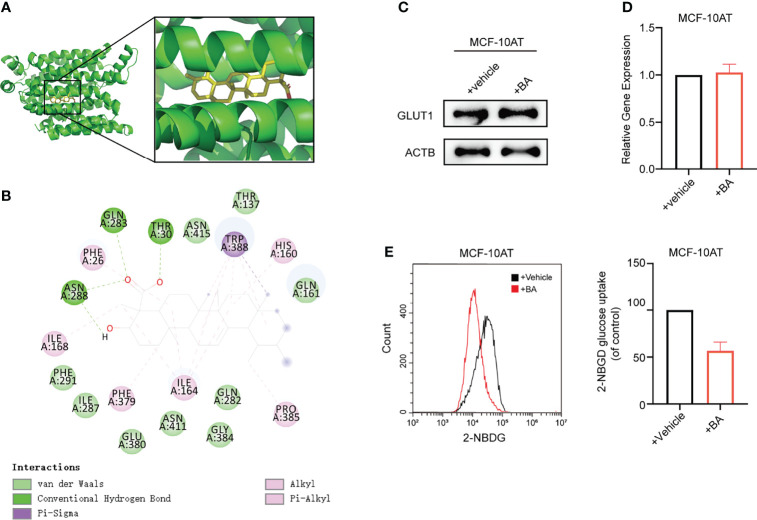
β-BA interacts with GLUT1 to block glucose uptake in MCF-10AT. **(A)** Visualization of 3D interaction of docked complex composed of GLUT1 and β-BA. **(B)** Ligand interactions between β-BA and GLUT1. **(C)** The protein expression of GLUT1 in MCF-10AT cell with or without β-BA treatment was measured by Western blot. **(D)** The gene expression of GLUT1 in MCF-10AT cell with or without β-BA treatment was measured by real-time PCR. **(E)** The 2-NBDG glucose uptake in GLUT1 overexpression MCF-10AT and its corresponding control cells was measured by FACS. Statistics are shown in the right panel as mean fluorescent intensity ± S.D.

To obtain further insight into the mechanisms of β-BA in regulating the glycolysis process, we used docking analysis to search potential targets of β-BA. To focus on the glycolysis process, we predicted the binding between β-BA and the key proteins in the glycolysis pathway. The molecular docking analysis was based on vital parameters like binding site and strength of molecular interactions. The initial structures were taken from the Protein Data Bank (PDB). Substrates were docked into the active site of protein using the AutoDock Vina tool (15) in Chimera (16). The docked poses with the highest docking scores were used for the subsequent studies. As a result, the highest affinity was reached between β-BA and GLUT1 (−10.5 kcal/mol), which was higher than the redocking score between natural substrate and GLUT1 (−6.9 kcal/mol) (**Table 1**). GLUT1 is a key transporter that regulates glucose absorbance and maintains intracellular glucose level, which governs the fuel of glycolysis. It was reported that Phe379, Trp388, and Trp412 played essential roles in GLUT1 function and glucose uptake (17–19). Notably, our results showed that β-BA bonded to the same pocket and interacted with these reported residues as natural substrate glucose (**Figures 4A, B**), which indicated that it might be a competent inhibitor for GLUT1. To validate the effect of β-BA on GLUT1, we checked the expression of GLUT1. As a result, neither the protein nor the mRNA level of GLUT1 in MCF-10AT was influenced following β-BA treatment (**Figures 4C, D**), while the results of 2-NBDG glucose uptake assay indicated that β-BA suppressed glucose transport (**Figure 4E**). In summary, β-BA could bind with GLUT1 to block glucose uptake, without influencing GLUT1 expression in MCF-10AT.

**Table 1:** Molecular docking scores (kcal/mol) of hit molecules with PDB IDs.

Ligand	Receptor	PDB ID	Affinity (kcal/mol)
β-BA	Aldolase	1QO5	−7.6
β-BA	Enolase	4ZA0	−8.6
β-BA	Glucose transporter 1	4PYP	−10.5
β-BA	Glyceraldehyde 3-phosphatedehydrogenase	1ZNQ	−8.4
β-BA	Hexokinase	2NZT	−8.3
β-BA	Lactate dehydrogenase	6SBV	−8.2
β-BA	Phosphoglycerate kinase	2X13	−8.0
β-BA	Phosphofructokinase l	4XYK	−6.2
β-BA	Phosphoglycerate mutase	5Y2I	−8.2
β-BA	Phosphohexose isomerase	6XUH	−5.6
β-BA	Pyruvate kinase	3ME3	−8.6
β-BA	Triose phosphate isomerase	4ZVJ	−8.1
Nonyl D-glucopyranoside	Glucose transporter 1	4PYP	

### 3.5 GLUT1 Mediates β-BA Regulated Glycolysis Suppression

**Figure 5:**
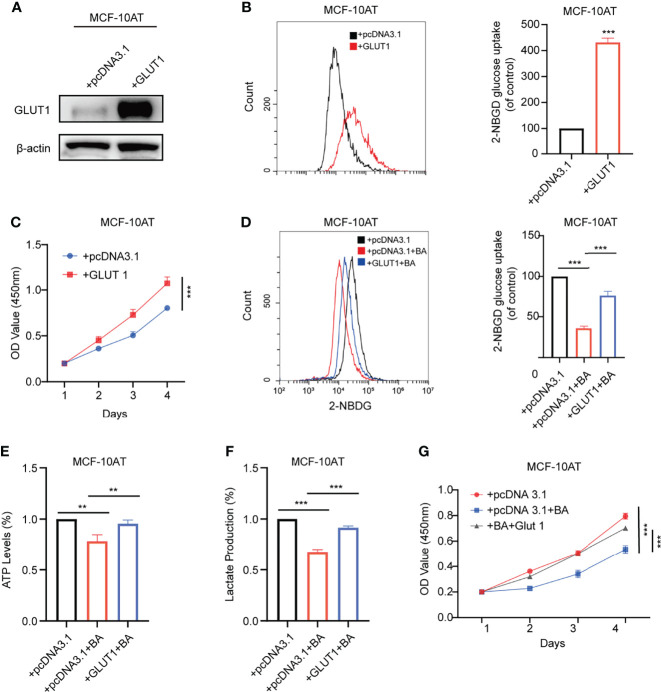
GLUT1 mediates β-BA regulated glycolysis suppression. **(A)** The overexpression level of GLUT1 protein was detected by Western blot. **(B)** The 2-NBDG glucose uptake levels of GLUT1 overexpressing and its corresponding control cells were detected by FACS. Statistics are shown in the right panel as mean fluorescent intensity ± S.D. **(C)** Cell viability was detected by CCK8 assay in GLUT1 overexpressing and its corresponding control cells. The glucose uptake level **(D)** ATP levels **(E)**, lactate production **(F)**, and cell viability **(G)** were measured in the group of MCF-10AT+pcDNA3.1, MCF-10AT+pcDNA3.1+BA, and MCF-10AT+GLUT1+BA, respectively. Data were shown as the mean ± S.D from independent experiments. **p < 0.01 and ***p < 0.001.

GLUT1 was reported to be upregulated in various types of carcinomas, while its function in breast precancerous cells remains to be elusive. We overexpressed GLUT1 by transfecting plasmid pcDNA3.1-GLUT1 into MCF-10AT and then tested the efficiency (**Figure 5A**). As shown in **Figures 5B, C**, forced expression of GLUT1 promoted cell proliferation and increased glucose uptake.

We further checked whether β-BA-regulated glycolysis alteration was related to GLUT1. The results suggested that overexpression of GLUT1 could predominantly reverse the proliferation inhibition induced by β-BA (**Figure 5G**). Consistently, overexpression of GLUT1 can also rescue the glycolysis phenotypes in MCF-10AT cells (**Figures 5D–F**). These results confirmed that GLUT1 was involved in β-BA-regulated glycolysis of MCF-10AT.

### 3.6 β-BA Prevented the Precancerous Lesion in Rat Disease Models

**Figure 6:**
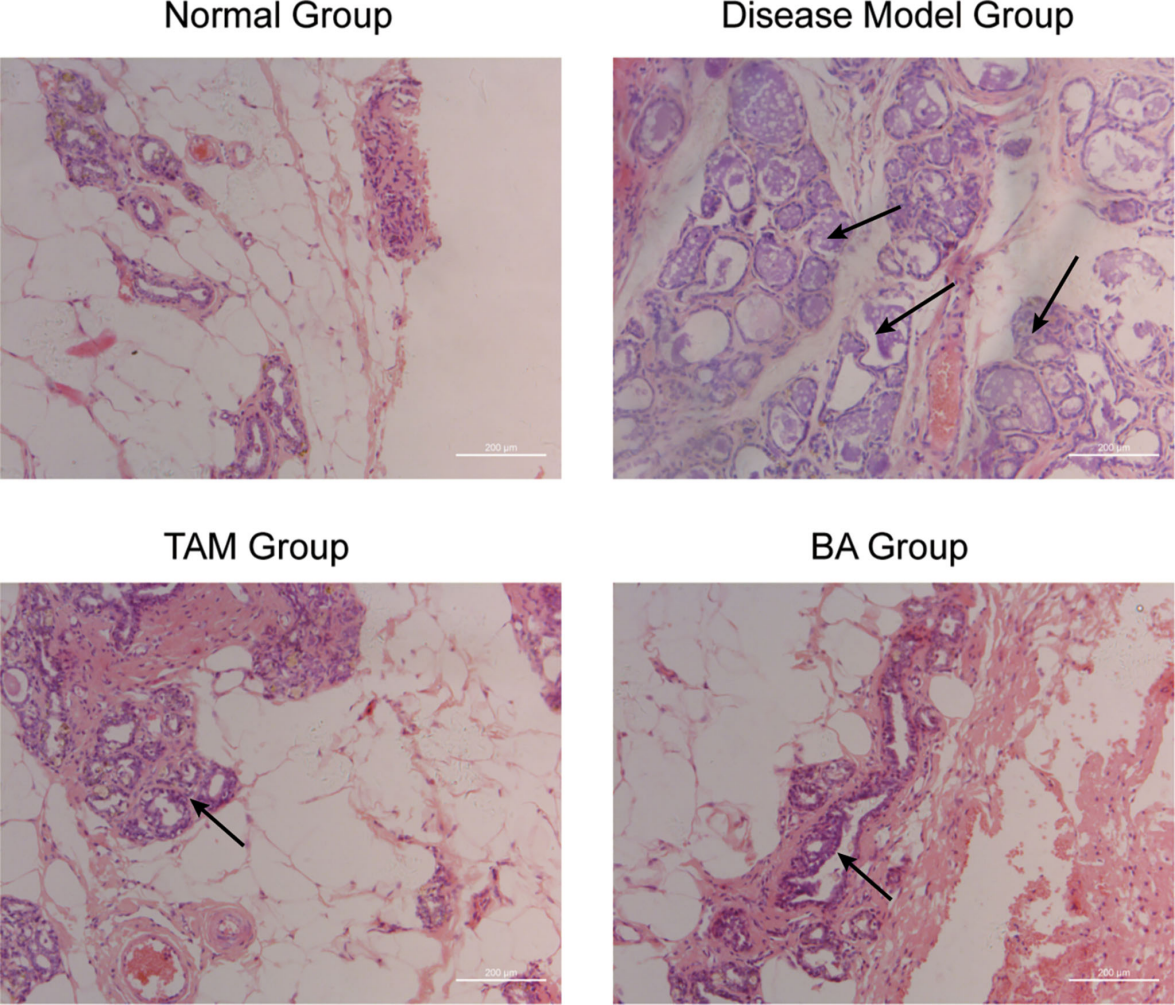
β-BA could inhibit the breast precancerous lesions *in vivo* This panel shows the hematoxylin–eosin staining of the control group, the disease model group, the tamoxifen-treated group, and the β-BA-treated group, respectively.

To further evaluate the therapy value of β-BA in breast precancerous prevention, β-BA was given to a precancerous rat model in the form of cream once a day. The animal model was established by DMBA combined with estrogen and progestin induction in SD female rats, with Tam used as a positive control. After 14 weeks of treatment, all the rats were sacrificed, and the morphology of mammary glands was checked by HE staining (**Figure 6**). As shown in **Table 2**, the rats in the normal group hardly showed abnormal hyperplasia, while in the model group, most animals developed atypical hyperplasia and invasive carcinoma (77.5%). After treating with β-BA cream, the dysplasia and carcinoma phenotypes were significantly prevented (44.2%), the effect of which was comparable to the Tam-positive treatment group (36.6%). These results further validated that β-BA could relieve precancerous breast lesion.

**Table 2:** The pathological changes of mammary gland tissue in rats in each group.

Groups	Breast number	No hyperplasia	General hyperplasia	Precancerous lesions	Invasive carcinoma
Normal	120	115	5	0	0
Model	120	0	27	86	7
Tam	120	13	63	42	2
β-BA	120	10	57	50	3

## 4 Discussion

Precancerous lesions mainly consist of atypical hyperplasia (atypical ductal or lobular hyperplasia), ductal carcinoma *in situ*, ductal carcinoma, and papillary tumor, which result in approximately 50% probability of deterioration (20). However, with positive intervention, the possibility of canceration may be reduced, while there is no consistent therapy for precancerous lesions.

Tam was approved by the FDA in 1978 for the treatment of ER-positive breast cancer and then further applied to reduce the incidence of breast cancer in high-risk women. An overview of the main outcomes in breast cancer prevention trials indicated about 30% to 40% reduction in ER-positive breast cancer incidence following 5-year Tam therapy versus placebo, while principal toxicities like endometrial cancers and thromboembolic events could not be avoided. Moreover, chemoresistance that appeared following Tam treatment may complicate the situation, which could be explained by the mutation and expression changes of ERa (21). In 2002, USPSTF announced that Tam was not suggested in non-high-risk populations unless risk and benefit were fully weighted. To avoid potential side effects, therapy targets should further focus on the significant differences between normal tissues and precancerous lesions. Our results indicated that β-BA-mediated glycolysis inhibition and subsequent proliferation suppression may play a positive role in breast precancerous prevention.

*B. serrata* as an herbal medicine has been widely used in folk medicine for centuries in Asian countries (like China, India, and Korea) to treat diverse disorders, such as inflammation, anxiety, and diabetes (22–26). BAs are the main active components of *B. serrata*. Several studies have proven that BAs could treat inflammation diseases and more importantly a variety of cancers. A study showed that topical application of Boswellin (a methanolic extract of *B. serrata*) could significantly inhibit skin inflammation, epidermal proliferation, and tumor promotion in the phorbol ester-induced rat model (27). A pre-clinical study reported that *B. serrata* extracts could alleviate the symptoms in patients with intracranial tumors while no side effects were observed during application (27). Many *in vitro* assays reported that BA could inhibit cell proliferation and induce apoptosis in cancer cells, including leukemic cells, melanoma cells, and glioblastoma cells (28–32). Our results suggested that β-BA could inhibit atypical hyperplasia in a rat model *in vivo*, and *in vitro* β-BA could suppress the proliferation and induce the apoptosis of MCF-10AT without a remarkable effect on MCF-10A. The detection of related proteins verified that β-BA could induce apoptosis in MCF-10AT but not in MCF-10A.

The Warburg effect is a well-known feature of various types of cancers. It is defined as an increase in the rate of glucose uptake and production of lactate anaerobically. It was found by Otto Warburg early in the 1920s, and extensively studied over the past 10 years to establish its causes and functions. Recently, some studies unveiled that due to its rapid conversion rate, the Warburg effect played an important role in biosynthesis and ATP production (33), while compared with the intensive interest in cancer, the function of the Warburg effect in precancerous tissues remains elusive. Lately, Chen et al. found that glucose consumption and lactate production increased in precancerous lesions through metabolomics analysis (5). In this work, we obtained possible targets of β-BA *via* the BATMAN-TCM website and submitted them for KEGG pathway enrichment. The results revealed that β-BA may exert an inhibition effect on MCF-10AT by targeting metabolism. Further metabolic-related assays suggested that β-BA suppressed glycolysis and ATP production. The AMPK pathway plays an important role in cell growth and metabolism as a fuel sensor and regulator. AMPK as a heterotrimeric complex is composed of a catalytic α-subunit and regulatory β- and γ-subunits. Cellular stress-induced AMP/ATP ratio increase activates AMPK by phosphorylating α-subunits. The activated AMPK will inhibit ATP-consuming pathways, like mTOR (34). The mammalian target of rapamycin (mTOR) is a central controller of cell growth and proliferation. There are two forms of mTOR complexes, mTOR complex 1 (mTORC1) and mTOR complex 2 (mTORC2). mTORC1, which is activated by multiple signals such as growth factors, amino acids, and cellular energy, regulates numerous essential cellular processes. It is reported that these two pathways serve as a signaling nexus for regulating cellular metabolism, energy homeostasis, and cell growth (35). Our study further revealed that β-BA-mediated glycolysis inhibition and ATP reduction would lead to AMPK α-subunit activation and mTOR pathway suppression, which might partly explain why β-BA could inhibit the proliferation and induce apoptosis on MCF-10AT.

The changes of glycolysis are often accompanied with alterations of related enzymes. To find the potential targets, we analyzed the interaction between β-BA and glycolytic enzymes *via* molecular docking. The highest score was reached between β-BA and GLUT1. Glucose transporter 1 (GLUT1) is a facilitative GLUT overexpressed in various types of tumors. It has been considered as an important target for cancer therapy. A previous study reported that the transformed mammary cells isolated from Glut1 conditional deletion mice grew slower in immunodeficient mice compared to control tumors (36). Another study presented that compared with the control breast cancer model group, even the Glut1 heterozygous group behaved like both allele deletion mice with occasional tumor development, which indicated the crucial role of Glut1 in breast tumor development (37). In this work, we identified β-BA to be a potential Glut1 inhibitor, which was not reported before. The 2-NBDG glucose uptake assay revealed that the combination of β-BA to Glut1 inhibited the intracellular glucose concentration and triggered acute metabolic stress. Although it has been reported that some small molecules derived from natural compounds could be GLUT1 inhibitors, most of them were not tested in actual disease models *in vivo*. In this article, we successfully constructed a breast precancerous model and proved that β-BA could suppress precancerous cell proliferation *in vivo*.

## 5 Conclusion

Overall, β-BA could suppress the glycolysis pathway and reduce ATP production in MCF-10AT cells without an obvious effect on normal MCF-10A. The induced metabolic stress activated the AMPK pathway and inhibited the mTOR pathway, which limited cell proliferation and promoted apoptosis. The consistent precancerous prevention effect of β-BA could be seen in the rat model. Mechanically, β-BA could combine with Glut1 and impair its glucose transporter function, which is critical for providing fuel to glycolysis. Taken together, β-BA is suggested to be a potential candidate for the treatment of breast precancerous lesions.

## Data Availability Statement

The original contributions presented in the study are included in the article/supplementary material. Further inquiries can be directed to the corresponding author.

## Ethics Statement

The animal study was reviewed and approved by the Laboratory Animal Ethics Committee of Jinan University. Written informed consent was obtained from the owners for the participation of their animals in this study.

## Author Contributions

FB and MM contributed to conception and wrote the article. GZ and XY performed the target prediction and KEGG enrichment analysis. XM and SZ performed *in vitro* experiments. YQ and JC performed animal experiments. YM analyzed the data and provided advice. All authors contributed to the article and approved the submitted version.

## Funding

This work was supported by the National Natural Science Foundation of China (nos. 82074430, 81803979, 81673979, 82073748, and 81741130); the Natural Science Foundation of Guangdong Province, China (nos. 2022A1515011674, 2018A030313393 and 2016A030313114); the Science and Technology Program of Guangzhou, China (nos. 201803010051, 201707010245, and 201704020117); the Fourth Batch of TCM Clinical Outstanding Talent Program of China (no. 444258); the Guangdong Basic and Applied Basic Research Foundation (nos. 2019A1515011866 and 2021A1515010993); the Guangdong Science and Technology Innovation Strategy Special fund (International Science and Technology Cooperation Projects, no. 2021A0505030034); and Jinan University’s National Collegiate Innovation and Startups Training Program (no. 202110559093). Thanks to the General Project of Natural Science Foundation of Guangdong Province, China in 2022.

## Conflict of Interest

The authors declare that the research was conducted in the absence of any commercial or financial relationships that could be construed as a potential conflict of interest.

## Publisher’s Note

All claims expressed in this article are solely those of the authors and do not necessarily represent those of their affiliated organizations, or those of the publisher, the editors and the reviewers. Any product that may be evaluated in this article, or claim that may be made by its manufacturer, is not guaranteed or endorsed by the publisher.

## Abbreviations

BA, boswellic acid; Tam, tamoxifen; DAVID, Database Visualization and Integrated Discovery; KEGG, Kyoto Encyclopedia of Genes and Genomes; AMPK, adenosine 5’-monophosphate (AMP)-activated protein kinase; mTOR, mammalian target of rapamycin; FBS, fetal bovine serum; USPSTF, the United States Preventive Services Task Force; ATCC, American Type Culture Collection; KCI, Karmanos Cancer Institute; ECAR, extracellular acidification rate; DMBA, 7,12-dimethyltetraphene.
